# Role of Dietary *Saccharomyces boulardii* in Innate Immune Responses of Broiler Chickens Fed Diets Containing Different Nutrient Densities

**DOI:** 10.3390/ani15233425

**Published:** 2025-11-27

**Authors:** Viet Anh Vu, Chreng Lis, Da-Hye Kim, Yong-Suk Lee, Kyung-Woo Lee

**Affiliations:** 1 Department of Animal Science and Technology, Konkuk University, 120 Neungdong-ro, Gwangjin-gu, Seoul 05029, Republic of Korea; vuvietanhpchy@gmail.com (V.A.V.); chrenglis@konkuk.ac.kr (C.L.); kdh142536@konkuk.ac.kr (D.-H.K.); 2 Eaglevet Co., Ltd., Yesan-Gun 32417, Republic of Korea; lysuk77@hanmail.net; 3 Animal Resources Research Center, Konkuk University, 120 Neungdong-ro, Gwangjin-gu, Seoul 05029, Republic of Korea

**Keywords:** *Saccharomyces boulardii*, nutrient density, growth, immune, broilers

## Abstract

The study was conducted to evaluate the influence of *Saccharomyces boulardii* (SB) supplementation on growth performance, meat quality, cecal volatile fatty acid (VFA) profile, immune parameters, and serum biochemistry in broiler chickens fed diets with varying nutrient densities. A total of 420 day-old male broiler chicks (Ross 308) were randomly allocated to 28 floor pens. A 2 × 2 factorial design was employed, with two factors: SB supplementation (none or 2.0 × 10^10^ CFU/ton), and optimal (OPT) and deficient nutrient density (DEF). The OPT diet significantly improved body weight gain and feed intake across all phases and enhanced the feed conversion ratio during the finisher and overall periods compared to the DEF diet (*p* < 0.05). However, SB supplementation decreased body weight gain during the starter and overall periods (*p* < 0.05). Serum levels of glutamic-oxaloacetic transaminase, glutamic-pyruvic transaminase, and triglyceride were elevated in chickens fed the OPT diet (*p* < 0.05). While SB supplementation did not affect meat quality or cecal VFA profiles, it interacted with nutrient density to influence alpha-1-acid glycoprotein and interferon-gamma concentrations in serum samples (*p* < 0.05). In conclusion, dietary supplementation with *S. boulardii* did not affect growth performance in broiler chickens regardless of nutrient density levels. However, it interacted with nutrient density to modulate the innate immune markers suggesting the immune-modulating role of *S. boulardii* in chickens.

## Simple Summary

Feed represents the largest share of broiler production costs, so the poultry industry aims to optimize nutrient density to support growth while minimizing costs. We hypothesized that *Saccharomyces boulardii*, a probiotic known for promoting gut health, could enhance growth performance in broiler chickens fed diets containing reduced nutrient densities. The experiment employed a 2 × 2 factorial design to test two factors: *Saccharomyces boulardii* supplementation (none or 2.0 × 10^10^ CFU/ton) and nutrient density (optimal or low). Results revealed that *Saccharomyces boulardii* supplementation failed to affect growth performance in broiler chickens fed on diets with different nutrient densities. However, dietary *S. boulardii* beneficially interacted with nutrient density to modulate the innate immune markers in broilers.

## 1. Introduction

Nutrient density in broilers’ diets plays a crucial role in their growth, health, and economic viability of production [1,2]. Studies suggest that broilers fed high-density starter diets exhibit improved growth throughout the rearing period [3]. High-density diets can potentially lead to reduced feed intake and an improved feed conversion ratio, resulting in increased carcass weight [4,5]. However, increasing nutrient density can concomitantly elevate feed costs, nitrogen excretion, fat deposition, and contribute to metabolic disorders in poultry [6]. On the other hand, Li et al. [7] observed that low-density diets reduced feed efficiency without impacting carcass yield, breast meat yield, thigh yield, or abdominal fat content. In addition to growth performance, dietary nutrient density can influence physiological responses related to immunity in broilers. Diets with restricted nutrient density could induce upregulation of immune-related genes in the chicken spleen and reduce the serum IgM level [8]. Nutrient restriction may also impair the antioxidant defense system, leading to increased oxidative stress that negatively affects growth, immune function, tissue integrity, and meat quality traits such as lipid oxidation and color stability [9,10]. Consequently, strategies that can alleviate oxidative stress and support immune responses are essential for sustaining performance and meat quality, especially under nutrient-restricted conditions.

In response to the limitations of suboptimal nutrient diets, research has explored feed additives as a nutritional strategy to improve the immunity and performance of broiler chickens [11]. Zanella et al. [12] reported that supplementing a low-nutrient-density diet with probiotics or exogenous enzymes enhanced growth performance and increased energy and amino acid digestibility in broilers. Similarly, Selim et al. [13] found that dietary supplementation of *S. cerevisiae* at 0.2% in a low non-phytate phosphorus diet improved the growth performance, health, nutrient digestibility, and bone quality of chickens.

The use of yeast as a natural growth promoter in livestock nutrition research has been extensively investigated for decades [14]. Beneficial effects of yeast supplementation in broilers were reported to increase growth performance [15] and enhance intestinal integrity and protection against pathogens [16]. The supplementation of yeast or yeast cell walls (*S. cerevisiae*) also increased meat tenderness and gut health indicators in broilers [17].

Among various yeast strains, *S. boulardii* (SB), a probiotic belonging to the *Saccharomyces* genus, has gained significant interest. First isolated from litchi fruit by Henri Boulard in the 1920s [18], *S. boulardii* possesses distinct physiological and metabolic characteristics compared to other strains. *S. boulardii* exhibits an optimal growth temperature of around 37 °C and demonstrates tolerance to low pH environments and high bile acid concentrations [19]. *S. boulardii* is a recently recognized probiotic strain known for its beneficial effect on intestinal health through modulation of the intestinal ultrastructure [20]. Moreover, it is a valuable protein source rich in amino acids and contributes to glutathione production which is an essential antioxidant [21]. Supplementation with SB has been shown to effectively modulate gut microbiota, promoting an increase in beneficial bacteria populations [22], and to exhibit positive effects on protein and globulin synthesis [23]. Kumari and Susmita [24] observed that probiotic SB at a concentration of 0.16 mg/L in drinking water improved performance and increased gut absorption capacity in broiler chickens. Furthermore, SB increased digestive enzyme activity in duodenal homogenates and augmented immune responses such as elevated production levels of immunoregulatory cytokines and increased numbers of immunoglobulin A (IgA) producing cells in broiler chickens [25]. However, information regarding the effect of SB on broilers fed diets containing different nutrient densities is relatively lacking. Thus, we attempted to investigate the beneficial role of probiotic supplementation in broilers fed on a low-nutrient-density diet. We aimed to evaluate the effect of probiotic SB on growth performance, meat quality, cecal volatile fatty acid, and immune and serum parameters under nutrient-restricted dietary conditions. We decided to use male chicks to lower potential bias by sex, if any, in responses to nutrient density and dietary SB.

## 2. Materials and Methods

### 2.1. Animal Care

All animal care procedures were approved by the Institutional Animal Care and Use Committee in Konkuk University (KU16138).

### 2.2. Animals, Diets, and Experimental Design

The experimental design was a 2 × 2 factorial arrangement with additives (none and SB at 2.0 × 10^10^ CFU/ton) and nutrient density (optimal and deficient). The optimal (OPT) or deficient nutrient density (DEF) diets (Table 1) were mixed with or without dietary SB to form four treatments: OPT diet without additive, DEF diet without additive, OPT diet plus SB, and DEF diet plus SB. This yeast used in this study contained 1.0 × 10^10^ CFU/kg of *Saccharomyces boulardii* CNCM I-1079 with a viability rate exceeding 99% [26]. Experimental diets in mash form were prepared on a weekly basis, but we did not attempt to isolate or monitor live *S. boulardii* CNCM I-1079 from the droppings.

A total of 420 day-old feather-sexed male broiler chicks (Ross 308) were purchased from a local hatchery, weighed individually upon arrival, and randomly housed into 28 floor pens, with 7 pens per treatment. Each pen measured 1 m × 2 m, had a pan feeder and nipples, and housed 15 birds. Feed and water were provided for ad libitum. Rice husk was used as a bedding material, and lighting was maintained for 23 h/d. The temperature was maintained at 32 °C during the first week and gradually decreased to 23 °C at 3 weeks and kept thereafter. The experiment lasted 5 weeks. No vaccinations were practiced in this study.

### 2.3. Growth Performance and Sample Collection

Body weight and feed intake per pen were recorded weekly and used to calculate the feed conversion ratio. Mortality was recorded daily and feed intake was corrected for mortality. At 35 days, one bird per pen close to average body weight was selected for sampling of the blood, small intestine, and ceca. Birds were euthanized by overdose of carbon dioxide. Immediately after euthanasia, blood was obtained in clot activator tubes by cardiac puncture. Serum samples were obtained by gentle centrifugation (200× *g*) for 15 min and stored at −20 °C before analysis. Then, right breast meat and the whole right leg (without skin) were sampled, weighted, and expressed as relative meat weight to the body weight. To measure secretory IgA, 5 cm-long segment of the jejunum proximal to the Meckel’s diverticulum was excised. Then, a pair of ceca was sampled for assaying volatile fatty acids.

### 2.4. Meat Quality of Breast and Leg Meat

The breast meats and thigh meats were used to measure the cooking loss, meat color, and pH at 24 h postmortem. The pH values of the breast and leg meat were measured in duplicate with a pH meter (Testo 205, Testo AG, Lenzkirch, Germany). Meat color was measured on the central side of the pectoralis major muscle in 3 different points using a portable spectrophotometer (CM-2600d, Konica Minolta, Ramsey, NJ, USA). The CIE lightness (L*), redness (a*), and yellowness (b*) components were obtained from the SCE mode readings. To measure the water-holding capacity, the right breast and deboned thigh meat were individually packaged in a plastic bag and cooked in a water bath at 80 °C for 30 min to reach an internal temperature of 70 °C, as described elsewhere [27]. After cooking, meat samples were cooled in ice cold water for 10 min to room temperature and residual moisture was removed with a paper towel before re-weighing. Cooking loss was calculated as the percentage of weight loss by the sample.

### 2.5. Gut Health Assay for Volatile Fatty Acid (VFA) and Jejunal Immunoglobulin a Contents

Approximately 0.5 g of cecal digesta was suspended in 4.5 mL of cold distilled water and mixed using a vortex mixer (C-VT Test Tube Voltex Mixer, Chang Shin Scientific Co, Incheon, Republic of Korea). The following were added to the mixtures: 0.05 mL of saturated HgCl_2_, 1 mL of 25% H_3_PO_4_, and 0.2 mL of 2% pivalic acid; they was centrifuged at 1000× *g* at 4 °C for 20 min, and the supernatant was used to measure the concentrations of VFA in cecal samples by gas chromatography (6890 Series GC System, HP, Palo Alto, CA, USA), as described by Kim et al. [28].

The jejunal segment was cut longitudinally and rinsed using ice-cold phosphate-buffered saline. The jejunal mucosa was obtained by gentle scraping of the jejunal segment with a sterile tissue culture scraper. sIgA concentrations in the jejunal mucosa were measured using quantitative chicken IgA ELISA immunoassay kits (Bethyl, Co., 25043 West FM 1097, Montgomery, TX, USA, Catalog No. E33-103), as described by the manufacturer’s recommendation. The amount of protein in the jejunal mucosa homogenates was determined using a bicinchoninic acid (BCA) protein assay kit (Thermo Scientific, Waltham, MA, USA). sIgA contents were expressed as μg sIgA per mg of total protein.

### 2.6. Serum Physiology for Antioxidant and Immune Parameters

Superoxide dismutase (SOD) concentration in serum samples was determined using a commercially available SOD determination kit (sigma, St. Louis, MO, USA). The concentrations of nitric oxide (NO) in serum samples were determined as described [29]. In brief, approximately an equal volume of serum samples and Griess reagent (Sigma, St. Louis, MO, USA) was added to each well in 96-well plate. The plate was incubated for 10 min at room temperature and read at the absorbance of 540 nm using a microtiter plate reader (Synergy2, BioTek, Winooski, VT, USA). The concentration of NO was calculated from the standard curve with sodium nitrite, as described elsewhere [29].

Alpha-1-acid glycoprotein (AGP) in the serum was determined using the Chicken Alpha-1-acid Glycoprotein Assay Kit (Life Diagnostics, Inc., West Chester, PA, USA). Serum IgA and IgM concentrations were determined using chicken-specific IgA and IgM ELISA quantitation kits (Bethyl Laboratories Inc., Montgomery, TX, USA). The IFN-γ level in the serum samples was quantified using chicken-specific IFN-γ kits (Cusabio Biotech, Newark, NJ, USA).

### 2.7. Serum Biochemical Parameters

Biochemical parameters including glutamic oxaloacetic transaminase (GOT), serum glutamic pyruvic transaminase (GPT), glucose, total cholesterol, triglyceride, phosphorus, and uric acid were analyzed by an automated biochemical analyzer (Fuji DRI-CHEM 7000i; Fujifilm Co., Tokyo, Japan).

### 2.8. Statistical Analysis

All data were analyzed using a mixed-model analysis of variance under a factorial arrangement of treatments with the PROC MIXED procedure of SAS (SAS 9.4, SAS Institute Inc., Cary, NC, USA). Each pen served as the experimental unit for all measurements. For proportional data such as mortality, a square-root transformed pen-level analysis was performed to meet the model’s assumptions. The statistical model included nutrient density (ND), additive (AD), and their interaction (ND × AD) as fixed effects, with the pen considered as a random effect. Data normality was assessed using the PROC UNIVARIATE procedure in SAS, which confirmed the absence of outliers. Treatment means were obtained using the LSMEANS statement, and the PDIFF option in SAS was applied to separate the means when significant differences were detected. Differences were considered statistically significant at *p* < 0.05.

## 3. Results

### 3.1. Growth Performance

The effects of nutrient density and feed additive on body weight gain, feed intake, and feed conversion ratio are presented in Table 2. Body weight gain was significantly increased in broiler chickens fed the OPT vs. DEF diets during the starter, finisher, and whole periods (*p* < 0.05). On the other hand, during the starter and whole periods, body weight gain was significantly decreased (*p* < 0.05) in the broilers fed the SB diet. No interaction between nutrient density and feed additive on body weight gain was observed (*p* > 0.05). Feed intake was significantly elevated in broilers fed the OPT diet compared to the DEF diet during both the starter and whole periods (*p* < 0.05). No significant differences in feed intake were observed by SB supplementation (*p* > 0.05). Moreover, an interaction between nutrient density and feed additive was not found (*p* > 0.05). Broiler chickens that consumed the OPT diet exhibited a significant improvement (*p* < 0.05) in feed conversion ratio during both the finisher and whole periods. Dietary SB failed to affect the feed conversion ratio (*p* > 0.05). Additionally, no interaction effect between nutrient density and feed additive on the feed conversion ratio was noted (*p* > 0.05). Mortality tended to be low (*p* = 0.076) in chickens fed the OPT vs. DEF diets although it remained low between treatment groups.

### 3.2. Meat Quality

The effects of nutrient density and feed additive on meat quality are presented in Table 3 and Table 4. None of the dietary treatments affected the relative weight, cooking loss, pH, L* value, a* value, and b* value of breast meats in boiler chickens (*p* > 0.05; Table 3). No interaction between the main factors on breast meat quality was observed (*p* > 0.05). Neither nutrient density nor feed additive significantly influenced thigh meat quality (*p* > 0.05; Table 4). There was no interactive effect between nutrient density and feed additive on thigh meat quality (*p* > 0.05).

### 3.3. Cecal Volatile Fatty Acid (VFA) Concentrations

Acetate was the dominant VFA followed by butyrate and propionate in cecal digesta (Table 5). The absolute concentrations of VFAs in the cecal digesta remained unchanged by nutrient density and feed additive treatments (*p* > 0.05). There was no significant interaction between main factors on cecal VFA concentration (*p* > 0.05).

### 3.4. Antioxidant Activity, Immune Parameters, and Serum Immunoglobulin Levels

The effects of nutrient density and feed additive on antioxidant and immune parameters and serum immunoglobulin levels are presented in Table 6. It is clear from this study that serum SOD levels at 35 days were not altered by dietary treatments (*p* > 0.05). Both nutrient density and feed additive failed (*p* > 0.05) to affect any of the immune parameters including jejunal sIgA levels, NO, and AGP at 35 days. No interactive effects by main factors (i.e., nutrient density and feed additive) were detected (*p* > 0.05) on serum SOD, NO, and jejunal sIgA levels. However, there were interactions between nutrient density and feed additive on AGP (alpha-1-acid glycoprotein) (*p* < 0.05). Broiler chickens fed an optimal nutrient density diet added with *S*. *boulardii* tended to have higher AGP levels by on average 37.5% compared with those fed a *S. boulardii*-free, optimal nutrient density diet. In contrast, adding *S. boulardii* into a deficient nutrient density diet significantly lowered AGP levels compared with those fed on a deficient nutrient density control diet.

Serum immunoglobulin and cytokine levels (IgA, IgM, and IFN-γ) remained unaffected by dietary treatments (i.e., nutrient density and feed additive) (*p* > 0.05). While there was no significant interaction between the main factors for concentrations of IgA and IgM in serum samples (*p* > 0.05), a significant interaction was noted between nutrient density and *S*. *boulardii* on IFN-γ production levels in broiler chickens (*p* < 0.05). When provided with optimal diets, birds supplemented with *S. boulardii* exhibited a non-significant increase by 10.6% in IFN-γ levels compared to the SB-free control group. Conversely, in broilers fed deficient nutrient diets, dietary *S. boulardii* led to a significant decrease in IFN-γ levels compared with the SB-free control diet-fed group.

### 3.5. Serum Parameters

Dietary supplementation with *S. boulardii* did not affect any of biochemical parameters (i.e., GOT, GPT, glucose, total cholesterol, triglyceride, phosphorus, and uric acid) (*p* > 0.05; Table 7). On the other hand, broilers fed the OPT vs. DEF diets exhibited significantly elevated serum levels of GOT, GPT, and triglyceride (*p* < 0.05). There were no interactive effects between the main factors on serum parameters (*p* > 0.05).

## 4. Discussion

The aim of this study was to investigate the effects of dietary *S. boulardii* on growth performance, meat quality, cecal VFA, and various markers in antioxidant, immunity, and biochemical physiology in broiler chickens fed diets containing different nutrient densities.

### 4.1. Effects of Nutrient Density

Nutrient density of the diet is a significant factor influencing broiler growth rate [30]. Broilers fed diets with the optimal nutrient density exhibited improved body weight gain and feed conversion ratio compared to those receiving deficient diets at all phases of production. These findings align with previous studies by Abdollahi et al. [31] and Kim et al. [32] who reported enhanced body weight gain, and Sun et al. [33] and Zhao et al. [2] who demonstrated an improved feed conversion ratio in broilers. Broilers fed optimal nutrient density diets also showed an increase in feed intake during the starter and whole periods. Majdeddin et al. [34] found a linear increase in feed intake with higher nutrient density during the grower and finisher periods. Increased feed consumption noted in this study was identified as the primary factor contributing to elevated body weight in chickens fed on optimal nutrient density diets. Previous research has established a positive correlation between nutrient density and body weight, where increased feed intake stimulated growth [6]. On the other hand, Delezie et al. [35] reported decreased feed intake of broilers fed on low metabolizable energy and crude protein diets. The reduction in feed intake observed in chickens fed low-nutrient-density diets might be likely attributed to decreased feed palatability.

The present study demonstrated that mortality rate tended to be higher in broilers fed deficient vs. optimal nutrient density diets, highlighting the critical role of dietary nutrient adequacy in promoting survival. These findings are consistent with recent studies, which have shown that diluted or suboptimal nutrient diets negatively affected productivity and increased the risk of mortality as chickens cannot fully compensate for their nutritional deficiencies by increasing feed intake [36,37]. Ensuring optimal nutrient density in broiler diets is therefore essential for improving production efficiency and reducing mortality risk in poultry production. Nonetheless, it should be remembered that mortality was kept low, being less than 3% across the treatment groups, indicating a minimal effect of nutrient density on the flock’s overall mortality in this study.

Serum biochemical markers are commonly employed for health monitoring, disease diagnosis, and assessing the nutritional status of chickens [38]. Key hepatic function indicators, such as GPT and GOT, also play a crucial role in amino acid metabolism [39]. In our study, we observed increased levels of GOT and GPT in groups receiving the OPT diet compared to those on the DEF diet. In contrast to our findings, low vs. optimal nutrient diets lowered [40] or did not affect [38] the GOT and GPT values in broiler chickens. Instead, Li et al. [41] suggested that the elevated levels of hepatic function indicators may be associated with the growth stage of broilers, during which higher GPT activity is required for glutamate synthesis, a process essential for muscle development. Thus, it is likely that the latter statement supports our findings.

The triglyceride concentration was elevated in OPT-diet-fed chickens compared to those on the DEF diet, indicating that the OPT diet may lead to elevated lipid levels. This result is similar to Choe et al. [42] who showed that triglycerides tended to rise with increased dietary energy, and Sigolo et al. [43] who reported that reducing dietary crude protein content decreased serum triglyceride concentrations. However, Jahanpour et al. [44] reported that birds exposed to severe feed restrictions for two weeks exhibited the highest triglyceride concentrations. Mohebodini et al. [45] also found higher triglyceride concentrations in the feed-restricted group. Furthermore, Swennen et al. [46] postulated that broilers fed on low protein diets could increase hepatic lipogenesis. Thus, our finding favors the increased availability of energy leading to higher lipogenesis in broilers fed the OPT vs. DEF diets. Further studies monitoring key enzymes (e.g., acetyl-coenzyme A carboxylase and fatty acid synthetase) in fatty acid metabolism would clarify the nutrient density-mediated increase in serum triglyceride levels.

### 4.2. Effect of Yeast Probiotic (S. boulardii)

*S. boulardii* is a prominent yeast probiotic within the *Saccharomyces* genus [47]. While it is primarily used in the food industry, *S. boulardii* has gained attention in animal nutrition for its potential benefits, including nutritional, metabolic, and antimicrobial properties [22]. In this study, we found that supplementation of yeast probiotic (SB at 2.0 × 10^10^ CFU/ton) failed to affect growth performance (i.e., feed intake and feed conversion ratio) in broiler chickens, similar to the previous finding of Rezaeipour et al. [48]. Moreover, these results were in accord with Mountzouris et al. [49], who reported that yeast probiotic was not effective in enhancing the growth performance of the birds. In the present study, dietary *S. boulardii* decreased body weight gain compared to the *S. boulardii*-free diet-fed control group. This discrepancy could be attributed to reduced, albeit marginal, feed intake by the *S. boulardii*-supplemented birds. Our findings agree with those of Abou El-Naga [50], who observed decreased feed intake in chickens fed a diet containing 0.5% dry yeast. Additionally, Gil de los santos et al. [51] reported that *S. boulardii*-supplemented diet-fed broilers ate 10% less than those fed on the control diet. However, our findings contradict other reports which demonstrated the positive influence of dietary *S. boulardii* on growth performance in broilers, as evidenced by improvements in body weight gain and feed conversion ratio [20,22].

Meat quality indices, including pH, color (lightness, redness, and yellowness), or cooking loss were measured commonly [52]. Meat color is an indicator of overall quality, with pale, soft, and exudative (PSE) meat causing economic concerns in the poultry industry [53]. The final pH of meat, established during postmortem biochemical reactions associated with rigor mortis, influences meat quality [54]. In our study, none of the breast and thigh meat qualities were influenced by dietary *S. boulardii*. It concurred with previous research by Aristides et al. [55] who observed no color differences in breast meats of broilers fed diets supplemented with *S. cerevisiae* components. In addition, Hoque et al. [56] reported no significant effects of probiotics on pH, water-holding capacity, and drip loss.

The concentration of cecal VFAs is widely recognized as a reliable indicator of gut health and also serves to inhibit the colonization of pathogenic bacteria [57]. Indeed, VFAs play a pivotal role in reducing *Enterobacteriaceae* populations in the ceca of broiler chickens [58]. Shanmugasundaram et al. [59] observed a reduction in *Salmonella* and *E. coli* populations (20% and 31%, respectively) in chickens fed diets supplemented with 0.2% yeast cell wall products. However, we found that dietary *S. boulardii* did not affect cecal VFAs. Our finding agrees with prior investigations that yeast-derived cell walls failed to influence cecal VFA concentrations in broiler chickens [60] and in turkey poults [61].

Among the antioxidative enzymes, SOD is one of the most important effector, playing the principle role of the self-defense mechanism [62]. In this study, levels of serum SOD did not differ among dietary treatments, which was in line with Rajput et al. [63], who noted the that SOD activities remained unaltered by the supplementation of *S. boulardii*. Secretory IgA is the dominant immunoglobulin in intestinal secretions and considers the effector of mucosal innate immunity [64]. It also plays a pivotal role in removing antigens from tissues via immune complex formation [65] and intraepithelial neutralization of virus replication [66]. Apparently, our study showed that dietary *S. boulardii* did not affect the sIgA levels in the jejunal mucosa of broiler chickens. Contrary to our findings, dietary *S. boulardii* improved sIgA levels in the duodenum [25] and total serum IgY levels in broilers [67].

We found that dietary *S. boulardii* did not alter any parameters of serum biochemical profiles, which is in agreement with the observation of Rajput et al. [63] who reported that serum levels of calcium, phosphorus, total protein, globulin, albumin and globulin ratio, and total and high-density lipoprotein cholesterol were not affected by dietary *S. cerevisiae*. On the other hand, dietary yeast decreased the serum concentrations of cholesterol and triglyceride concentrations, but increased serum concentrations of protein and albumin in broiler chickens [68,69].

A possible explanation for the lack of beneficial effects observed in our study might be related to the viability or activity of *S. boulardii* within the gastrointestinal tract. Although *S. boulardii* has demonstrated good viability in simulated gastric and intestinal fluids [19], we did not attempt to isolate *S. boulardii* in fecal droppings to monitor its viability. Despite its potential to transiently colonize the gut, it is known that competitive exclusion exerted by the indigenous microbiota may also restrict colonization of probiotic *S. boulardii* [70]. However, it should be remembered that all experimental diets, including probiotic *S. boulardii*, were mixed on a weekly basis to minimize, if any, the loss of viability in feeds. In any event, further assessment of yeast recovery from intestinal digesta or feces is necessary to confirm survival and colonization ability.

Another important consideration is the strain-specific variation in probiotic properties. Although all strains are classified as *S. boulardii*, considerable variation exists in their metabolic activity, resistance to gastrointestinal stress, and immunomodulatory properties. The comparative study of commercial *S. boulardii* strains has demonstrated differences in techno-functional traits, including auto-aggregation, cell surface hydrophobicity, survival under low pH and bile salt exposure, and antimicrobial activity against pathogens, which influence their survival and the viable dose delivered to the intestine [70,71]. These differences can lead to variable physiological effects in the host and help explain inconsistencies reported across studies.

### 4.3. Interaction Between Yeast Probiotic (S. boulardii) and Nutrient Density

α1-acid glycoprotein (AGP) is an acute phase protein in chicks, reflecting innate immune activation [72]. Interferon-gamma (IFN-γ) is a crucial cytokine involved in host defense against infections, regulating inflammatory responses [73]. In this study, we found that nutrient density and dietary *S. boulardii* exhibited an interaction on AGP and IFN-γ levels in serum samples. Although supplementation of dietary *S. boulardii* tended to increase both AGP and IFN-γ levels in broilers fed on the OPT diets, the statistical significances were not noted. However, dietary *S. boulardii* significantly lowered both AGP and IFN-γ levels in broilers fed DEF, but not OPT, diets, leading to significant interaction. Thus, it seems apparent that dietary *S. boulardii* modulated innate immune responses (e.g., AGP and IFN-γ) in broilers fed on the suboptimal nutrient diet. Our findings do indicate that dietary *S. boulardii* tended to augment innate responses under optimal nutrition environments but can mitigate a deficient nutrition density diet-induced increase in innate immune response in broiler chickens.

Nutrient unbalance or deficiency in early broiler chicks has been associated with metabolic changes that impair systemic and local immune competence due in part to reduced lymphoid organ growth [74]. Yu et al. [75] highlighted in their review that unbalanced nutrition in the diets of chickens could increase inflammation and decrease immune functions, including antibody production, and dietary functional nutrients, including probiotics, can restore once-disrupted immune balance by nutritional deficiency (or imbalance), stress, or pathogen exposure. Furthermore, it is documented that low vs. high dietary protein levels could induce interleukin (IL)-1 production in broiler chickens [76]. Previous studies have reported that dietary *S. boulardii* can suppress the secretion of proinflammatory cytokines (i.e., IL-6, tumor necrosis factor-α [TNF-α], and IL-1β) induced by deoxynivalenol in porcine alveolar macrophage cells (PAMCs) [77]. Additionally, Lee et al. [78] found that dietary *S. boulardii* downregulated the expression of proinflammatory cytokines (i.e., IL-1β and TNF-α) in rats with trinitrobenzenesulphonic acid-induced colitis. Kühle et al. [79] reported that *S. cerevisiae* var. *boulardii* decreased the expression of proinflammatory IL-1α in *E. coli*-infected cells. These results suggest that dietary *S. boulardii* may exhibit superior anti-inflammatory abilities in nutritionally compromised chickens, leading to a significant decrease in innate immune markers (i.e., AGP and IFN-γ). It is known that stress can trigger inflammatory responses, and the upregulation of IL-10 may serve as a protective mechanism against oxidative stress-induced cellular damage [80]. Thus, it is likely that dietary *S. boulardii* could effectively enhance the IL-10 levels leading to the suppression of IFN-γ production in chickens fed the deficient nutrient diets. It is, however, kept in mind that different yeast species or products could induce different immunomodulatory effects in broilers [81]. Although AGP and IFN-γ, as the innate effector molecules, provide useful information on systemic inflammation and Th1-type immune responses, they do not comprehensively represent the cytokine network involved in intestinal immune regulation. Analyzing additional cytokines such as IL-6, TNF-α, and IL-10 in future investigations would allow for a more detailed understanding of the immune-modulating capacity of *S. boulardii*, particularly in response to dietary nutrient density. In any event, our study suggests that dietary *S. boulardii* could modulate innate immune responses in broiler chickens under nutrition-induced stress environments.

## 5. Conclusions

The present study showed that the OPT vs. DEF diets positively influenced performance of broiler chickens at all phases of production. The OPT vs. DEF diets also elevated serum levels of GOT, GPT, and triglyceride. Dietary *S. boulardii* failed to affect growth performance in broiler chickens regardless of nutrient density levels. The primary finding of this study is that the dietary *S. boulardii* modulated innate markers (i.e., α1-acid glycoprotein and interferon-gamma levels) in serum samples and its immune-modulatory effect was dependent on nutrient density.

## Figures and Tables

**Table 1:** Ingredients and nutrient composition of the experimental diets ^1^.

	Starter (0–21 d)		Finisher (22–35 d)	
Item	OPT	DEF	OPT	DEF
Ingredients (g/kg)				
Maize, 8.8% CP	570	580	636.5	646.5
Soybean meal, 44.6% CP	320	320	255	255
Corn gluten meal, 60% CP	40	10	40	10
Soybean oil	25	25	30	30
Salt	3	3	2.4	2.4
Dicalcium phosphate	17	17	13	13
DL-methionine, 99%	3.1	3.1	2.0	2.0
L-lysine, 78%	2.0	2.0	2.2	2.2
L-threonine	0.5	0.5	0.5	0.5
Limestone	13	13	13	13
Sodium bicarbonate	2.4	2.4	1.4	1.4
Choline chloride, 50%	2.0	2.0	2.0	2.0
Cellulose	0.0	20.0	0.0	20.0
Vitamin premix ^2^	1.0	1.0	1.0	1.0
Mineral premix ^3^	1.0	1.0	1.0	1.0
Total	1000.0	1000.0	1000.0	1000.0
Nutrient composition ^4^ (%)				
AMEn (kcal/kg)	3049	2971	3152	3074
Dry matter	89.1	89.5	89.3	89.5
Crude protein	22.1	20.4	19.8	18.1
Total lysine	1.26	1.23	1.10	1.08
Methionine	0.65	0.61	0.52	0.48
Total TSAA	1.00	0.93	0.84	0.76
Total threonine	0.87	0.81	0.77	0.72
Calcium	1.00	1.00	0.9	0.9
Nonphytate phosphorus	0.45	0.45	0.37	0.37

^1^ OPT, optimal nutrient density; DEF, deficient nutrient density (in energy, crude protein and lysine); CP, crude protein; AMEn, nitrogen-corrected apparent metabolizable energy; TSAA, total sulfur amino acid. ^2^ Vitamin premix provided following nutrients per kg of diet: vitamin A, 12,000 IU; vitamin D3, 3000 IU; vitamin E, 40 IU; vitamin K3, 2 mg; vitamin B1, 2 mg; vitamin B2, 5 mg; vitamin B6, 3 mg; vitamin B12, 0.02 mg; niacin, 40 mg; biotin, 0.15 mg; pantothenic acid, 10 mg. ^3^ Mineral premix provided following nutrients per kg of diet: Fe, 88 mg; Mn, 66 mg; Zn, 60 mg; I, 0.99 mg; Se, 0.22 mg; Cu, 72.6 mg; Co, 0.33 mg. ^4^ Calculated values.

**Table 2:** Effect of nutrient density and feed additive on growth performance and mortality of broilers ^1^.

		Body Weight Gain, g/d/Bird			Feed Intake, g/d/Bird			Feed Conversion Ratio			Mortality (%)
Density	*S. boulardii*	1–21 d	21–35 d	1–35 d	1–21 d	21–35 d	1–35 d	1–21 d	21–35 d	1–35 d	
OPT	−	39.58 ^2^	78.46	55.13	57.71	133.5	88.02	1.39	1.71	1.51	0.952
	+	36.43	79.34	53.59	57.20	131.3	86.84	1.46	1.66	1.54	0.000
DEF	−	36.31	74.99	51.79	55.57	129.1	84.97	1.46	1.73	1.57	2.857
	+	35.74	70.16	49.51	54.36	128.9	84.17	1.43	1.84	1.59	1.905
Pooled SEM		0.771	2.011	0.794	0.822	2.029	1.080	0.031	0.039	0.017	0.273
Main factors											
OPT		38.00 ^a^	78.90 ^a^	54.36 ^a^	57.46 ^a^	132.4	87.43 ^a^	1.42	1.68 ^b^	1.53 ^b^	0.317
DEF		36.02 ^b^	72.58 ^b^	50.65 ^b^	54.96 ^b^	129.0	84.57 ^b^	1.45	1.78 ^a^	1.58 ^a^	2.222
	−	37.95	76.73	53.46 ^A^	56.64	131.3	86.50	1.43	1.72	1.54	1.905
	+	36.08	74.75	51.55 ^B^	55.78	130.1	85.51	1.45	1.75	1.57	0.952
*p*-value											
Density (ND)		0.017	0.004	<0.0001	0.006	0.106	0.014	0.465	0.018	0.004	0.076
Additive (AD)		0.024	0.336	0.024	0.302	0.569	0.369	0.525	0.393	0.138	0.364
ND × AD		0.109	0.169	0.644	0.674	0.624	0.860	0.094	0.054	0.975	1.000

^1^ OTP, optimal nutrient density; DEF, deficient nutrient density; SEM, standard error of the means. ^2^ Values are LS means of 7 replicates per treatment. ^a,b; A,B^ Values having a different superscript within a column differ significantly (*p* < 0.05).

**Table 3:** Effect of nutrient density and feed additive on breast meat quality of broilers ^1^.

		Breast Meat (g/100 BW)	Cooking Loss (%)	pH	CIE L* (Lightness)	CIE a* (Redness)	CIE b* (Yellowness)
Density	*S. boulardii*						
OPT	−	7.525 ^2^	27.77	6.056	55.27	0.484	11.82
	+	7.217	27.98	6.026	54.00	0.190	11.35
DEF	−	7.616	25.66	6.002	54.09	−0.121	9.774
	+	7.351	28.56	6.090	55.74	0.089	10.81
Pooled SEM		0.236	0.982	0.113	1.356	0.363	0.789
Main factors							
OPT		7.371	27.87	6.041	54.63	0.337	11.59
DEF		7.483	27.11	6.046	54.92	−0.016	10.29
	−	7.571	26.71	6.029	54.68	0.181	10.80
	+	7.284	28.27	6.058	54.87	0.139	11.08
*p*-value							
Density (ND)		0.639	0.443	0.968	0.837	0.340	0.114
Additive (AD)		0.237	0.126	0.800	0.890	0.909	0.724
ND × AD		0.927	0.183	0.606	0.290	0.494	0.352

^1^ OTP, optimal nutrient density; DEF, deficient nutrient density; SEM, standard error of the means. ^2^ Values are LS means of 7 replicates per treatment.

**Table 4:** Effect of nutrient density and feed additive on thigh meat quality of broilers ^1^.

		Thigh Meat (g/100 BW)	Cooking Loss (%)	pH	CIE L* (Lightness)	CIE a* (Redness)	CIE b* (Yellowness)
Density	*S. boulardii*						
OPT	−	6.908 ^2^	33.66	5.602	52.89	3.133	12.15
	+	6.748	33.19	5.633	55.51	3.137	12.95
DEF	−	6.381	35.24	5.664	55.81	2.541	11.42
	+	6.679	34.66	5.573	54.96	2.687	11.70
Pooled SEM		0.168	1.400	0.038	1.266	0.410	0.543
Main factors							
OPT		6.828 ^3^	33.42	5.618	54.20	3.135	12.55
DEF		6.530	34.95	5.619	55.38	2.614	11.56
	−	6.645 ^4^	34.45	5.633	54.35	2.837	11.78
	+	6.714	33.92	5.603	55.24	2.912	12.32
*p*-value							
Density (ND)		0.089	0.286	0.978	0.360	0.216	0.081
Additive (AD)		0.685	0.712	0.431	0.492	0.856	0.330
ND × AD		0.187	0.968	0.120	0.184	0.864	0.635

^1^ OTP, optimal nutrient density; DEF, deficient nutrient density; SEM, standard error of the means. ^2^ Values are least square means of 7 replicates per treatment. ^3^ Values are least square means of 14 replicates per treatment. ^4^ Values are least square means of 14 replicates per treatment.

**Table 5:** Effect of nutrient density and feed additive on concentrations (μM/g or % total) of volatile fatty acid (VFA) in cecal digesta of broilers ^1^.

Density	*S. boulardii*	Acetate	Propionate	Butyrate	Total VFA
μM/g					
OPT	−	30.15 ^2^	9.083	9.256	48.48
	+	33.80	9.369	12.27	55.44
DEF	−	30.36	9.410	9.435	49.20
	+	33.69	9.097	11.23	54.02
Pooled SEM		3.447	1.120	18.96	4.882
Main factors					
OPT		31.97	9.226	10.76	51.96
DEF		32.03	9.253	10.33	51.61
	−	30.25	9.246	9.35	48.84
	+	33.75	9.233	11.75	54.73
*p*-value					
Density (ND)		0.988	0.981	0.985	0.943
Additive (AD)		0.321	0.991	0.863	0.240
ND × AD		0.964	0.792	0.988	0.829
% of total VFA					
OPT	−	60.15	21.95	17.90	
	+	60.86	17.14	21.99	
DEF	−	61.77	19.73	18.50	
	+	61.93	17.65	20.42	
Pooled SEM		1.947	2.883	3.208	
Main factors					
OPT		60.51	19.55	19.95	
DEF		61.85	18.69	19.46	
	−	60.96	20.84	18.20	
	+	61.40	17.40	21.21	
*p*-value					
Density (ND)		0.498	0.769	0.881	
Additive (AD)		0.824	0.244	0.358	
ND × AD		0.889	0.640	0.737	

^1^ OTP, optimal nutrient density; DEF, deficient nutrient density; SEM, standard error of the means. ^2^ Values are LS means of 7 replicates per treatment.

**Table 6:** Effect of nutrient density and feed additive on superoxide dismutase (SOD) activity, immune parameter, and serum immunoglobulin levels of broilers ^1^.

		SOD Activity	Jejunal sIgA (μg/mg of Protein)	NO (μmol/L)	AGP (μg/mL)	IgA (mg/mL)	IgM (mg/mL)	IFN-γ (pg/mL)
Density	*S. boulardii*							
OPT	−	72.42 ^2^	16.998	18.76	366.1 ^ab^	1.190	1.657	119.3 ^ab^
	+	77.46	17.455	17.49	503.2 ^a^	1.094	1.533	131.9 ^ab^
DEF	−	74.41	3.959	19.87	491.9 ^a^	1.153	1.505	185.2 ^a^
	+	70.29	4.046	21.09	210.2 ^b^	1.146	1.345	101.4 ^b^
Pooled SEM		7.855	0.806	2.114	62.28	0.056	0.096	20.95
Main factors								
OPT		74.94	3.067	18.13	434.6	1.142	1.595	125.6
DEF		72.35	4.003	20.48	351.0	1.149	1.425	143.3
	−	73.41	3.908	19.32	429.0	1.171	1.581	152.2
	+	73.88	3.162	19.29	356.7	1.120	1.439	116.7
*p*-value								
Density (ND)		0.745	0.267	0.276	0.202	0.896	0.088	0.406
Additive (AD)		0.953	0.374	0.990	0.267	0.381	0.152	0.102
ND × AD		0.565	0.322	0.562	0.003	0.447	0.853	0.030

^1^ OPT, optimal nutrient density; DEF, deficient nutrient density; sIgA, secretory immunoglobulin A; NO, nitric oxide; AGP, alpha-1-acid glycoprotein; IgA, immunoglobulin A; IgM, immunoglobulin M; IFN-γ, interferon-gamma; SEM, standard error of the means. ^2^ Values are LS means of 7 replicates per treatment. ^a,b^ Values having a different superscript within a column differ significantly (*p* < 0.05).

**Table 7:** Effect of nutrient density and feed additive on serum parameters of broilers ^1^.

		GOT (IU/L)	GPT (IU/L)	Glucose (mg/dL)	Total Cholesterol (mg/dL)	Triglyceride (mg/dL)	P (mg/dL)	Uric Acid (mg/dL)
Density	*S. boulardii*							
OPT	−	175.4 ^2^	3.86	413.7	83.00	146.9	11.29	7.600
	+	195.9	4.29	404.4	87.29	146.6	11.69	7.686
DEF	−	156.6	3.57	376.1	81.43	85.43	10.64	6.743
	+	166.1	2.71	257.3	79.43	43.71	9.586	4.529
Pooled SEM		11.73	0.373	48.29	4.709	36.55	0.817	1.124
Main factors								
OPT		185.6 ^a^	4.071 ^a^	409.1	85.14	146.7 ^a^	11.49	7.643
DEF		161.4 ^b^	3.143 ^b^	316.7	80.43	64.57 ^b^	10.11	5.636
	−	166.0	3.714	394.9	82.21	116.1	10.96	7.171
	+	181.0	3.500	330.9	83.36	95.14	10.64	6.107
*p*-value								
Density (ND)		0.049	0.020	0.068	0.327	0.034	0.106	0.087
Additive (AD)		0.213	0.571	0.197	0.810	0.571	0.691	0.353
ND × AD		0.648	0.098	0.268	0.511	0.576	0.381	0.317

^1^ OPT, optimal nutrient density; DEF, deficient nutrient density; GOT, glutamic oxaloacetic transaminase; GPT, glutamic pyruvate transaminase; P, phosphorus; SEM, standard error of the means. ^2^ Values are LS means of 7 replicates per treatment. ^a,b^ Values having a different superscript within a column differ significantly (*p* < 0.05).

## Data Availability

The original contributions presented in this study are included in the article. Further inquiries can be directed to the corresponding author.
